# Investigating the Relationship between Epigenetic Age and Cardiovascular Risk in a Population with Overweight/Obesity

**DOI:** 10.3390/biomedicines12081631

**Published:** 2024-07-23

**Authors:** Davide Marinello, Chiara Favero, Benedetta Albetti, Davide Barbuto, Luisella Vigna, Angela Cecilia Pesatori, Valentina Bollati, Luca Ferrari

**Affiliations:** 1 EPIGET LAB, Department of Clinical Sciences and Community Health, Dipartimento di Eccellenza 2024–2027, University of Milan, 20122 Milan, Italy; 2 Occupational Health Unit, Fondazione IRCCS Ca’ Granda Ospedale Maggiore Policlinico, 20122 Milan, Italy

**Keywords:** epigenetic age, age acceleration, obesity, cardiovascular risk, Framingham risk score

## Abstract

**Introduction**: Cardiovascular diseases stand as the leading global cause of mortality. Major modifiable risk factors encompass overweight/obese conditions, high blood pressure, elevated LDL cholesterol, diabetes, smoking, secondhand smoke exposure, unhealthy diet, and physical inactivity. In the present study, we explored the relationship between cardiovascular risk factors and epigenetic age (DNAm age), an estimate reflecting an individual’s actual physiological functionality and overall health. Additionally, we assessed the association between DNAm age acceleration and cardiovascular risk, as evaluated through the Framingham risk score (FRS). **Methods**: The study includes 190 subjects with overweight/obese conditions. We calculated their DNAm age using Zbieć-Piekarska et al.’s DNAm age estimator on five sets of CpGs analyzed in the peripheral leucocytes. Linear regression models were employed to test the associations. **Results**: Various parameters contributing to increased cardiovascular risk were associated with DNAm age acceleration, such as systolic blood pressure (β = 0.045; SE = 0.019; *p* = 0.019), heart rate (β = 0.096; SE = 0.032; *p* = 0.003), blood glucose (β = 0.025; SE = 0.012; *p* = 0.030), glycated hemoglobin (β = 0.105; SE = 0.042; *p* = 0.013), diabetes (β = 2.247; SE = 0.841; *p* = 0.008), and menopausal conditions (β = 2.942; SE = 1.207; *p* = 0.016), as well as neutrophil (β = 0.100; SE = 0.042; *p* = 0.018) and granulocyte (β = 0.095; SE = 0.044; *p* = 0.033) counts. Moreover, DNAm age acceleration raised the FRS (∆% 5.3%, 95% CI 0.8; 9.9, *p* = 0.019). **Conclusion**: For the first time, we report that cardiovascular risk factors accelerated DNAm age in a selected population of hypersusceptible individuals with overweight or obesity. Our results highlight the potential of DNAm age acceleration as a biomarker of cumulative effects in cardiovascular risk assessment.

## 1. Introduction

Cardiovascular diseases (CVDs) stand as the primary global cause of mortality, contributing to around 18.6 million deaths worldwide annually [1]. Overweight and obesity, defined as a body mass index (BMI) of ≥25 and ≥30 kg/m^2^, respectively, is strongly linked to an elevated cardiovascular risk. Other major modifiable risk factors for CVDs include high blood pressure, high low-density lipoprotein (LDL) cholesterol, diabetes, smoking and secondhand smoke exposure, unhealthy diet, and physical inactivity [2]. It is not surprising that these factors are all associated with CVD because they lead to the same biological effects: low-grade systemic inflammation, redox imbalance, mitochondrial dysfunction, the accumulation of cytotoxic macromolecules, and impairment of the immune system, all mechanisms with a crucial role in the pathophysiology of CVD [2,3].

Age is an independent unmodifiable risk factor for CVD, and aging is a physiological process associated with a progressive deterioration in cardiovascular function [4]. Notably, chronological age does not always correspond to biological age, referring to an individual’s age as determined by his/her overall health, physiological condition, and actual cell functionality [5]. Therefore, it is increasingly evident that it is crucial to evaluate the role of biological age in cardiovascular risk assessment, especially in high-susceptibility conditions, such as obesity.

Epigenetic modifications such as DNA methylation are hallmarks of biological aging [6]. Over the past decade, a variety of models have been developed for the estimation of epigenetic age (DNAm age) through so-called epigenetic clocks, which are based on patterns of DNA methylation [7]. Among the most prominent clocks, the Hannum Clock focuses on 71 CpG sites associated with age-related changes in the blood cells [8]. The Horvath Clock incorporated 353 CpGs and was designed across multiple tissues, including sites from the Hannum Clock for blood cells, as a potential “pan-tissue” master clock for DNAm age [9]. Starting from these first-generation clocks, different minimized clocks have been developed that incorporate only a limited number of CpG sites strongly correlated with biological age. One notable example is the DNAm age estimator proposed by Zbieć-Piekarska and colleagues, which is based on five sets of CpGs analyzed in the peripheral leukocytes and consistently shows a strong correlation with chronological age [10,11,12].

Under physiological conditions, DNAm age is expected to align with chronological age, meaning there is no DNAm age acceleration, referred to as the residue between chronological age and DNAm age. However, in pathological conditions such as obesity, there have been reports of DNAm age acceleration, suggesting an increased risk of age-related conditions like CVD [3].

In recent years, several models have been developed for estimating cardiovascular risk based on various risk factors to which individuals have been exposed. Among them, the Framingham risk score (FRS), developed based on studies from the Framingham Heart Study, allows for the estimation of an individual’s risk of developing CVD, such as coronary heart disease or stroke, over 10 years [13].

In the present study, within the context of overweight/obesity—a major risk factor for CVDs—we aimed to elucidate the link between cardiovascular risk factors and DNAm age. We estimated DNAm age using the estimator proposed by Zbieć-Piekarska. Additionally, we investigated the association between DNAm age acceleration and cardiovascular risk, which was assessed using the FRS.

## 2. Materials and Methods

### 2.1. Study Population, Personal Data, and Biological Samples

The study was carried out on subjects recruited between 2010 and 2015 at the Center for Obesity and Work (Department of Preventive Medicine, IRCCS Fondazione Cà Granda Ospedale Maggiore Policlinico at the University of Milan). We randomly selected a subgroup of 190 people already enrolled in the context of the larger study SPHERE (ERC-2011-STG 282413) [14].

The study was approved by the Ethics Committee of the Fondazione Cà Granda-Ospedale Maggiore Policlinico (Approval no.1425), following the Declaration of Helsinki’s principles. Each participant who agreed to participate signed a written informed consent and subsequently was asked to complete lifestyle and dietary questionnaires, including on their current and past smoking habits, alcohol consumption, and physical activity.

According to the European Association for the Study of Obesity (EASO), we have committed to minimizing the bias related to the term “obesity” as much as possible and eliminating the stigma attached to labeling individuals based on their condition [15]. The eligibility criteria for enrolment in the study, as previously described [16], were (1) being older than 18 years at enrolment; (2) being overweight/obese according to the following definition: overweight is defined as a BMI between 25 and 30 kg/m^2^ and obesity is defined as a BMI of 30 kg/m^2^ or more; (3) being resident in Lombardy at the time of the recruitment; and (4) agreement to signing informed consent and donating blood samples. The exclusion criteria included a previous diagnosis of cancer, heart disease, or stroke in the last year or of other chronic diseases, such as multiple sclerosis, Alzheimer’s disease, Parkinson’s disease, depression, bipolar disorder, schizophrenia, or epilepsy.

### 2.2. Sample Collection and DNA Extraction

Seven mL of whole blood from each participant was collected into ethylenediaminetetraacetic acid (EDTA) tubes by venous phlebotomy. The blood was centrifuged at 2500 rpm for 15 min. The buffy coat fraction was transferred into cryovials and immediately frozen at −80 °C until use. Genomic DNA was extracted from 250 µL of the buffy coat using the Wizard^®^ Genomic DNA Purification Kit (Promega, Medison, WI, USA), according to the manufacturer’s instructions.

### 2.3. Bisulphite Conversion 

Genomic DNA (500 ng) was bisulphite-converted using the EZ DNA Methylation Direct Kit (Zymo Research, Orange, CA, USA), in accordance with the manufacturer’s protocol. The converted DNA was eluted in 30 µL of M-Elution Buffer. 

### 2.4. Determination of Epigenetic Age

DNAm age was calculated considering the methylation pattern of five CpG sites at five genes (*ELOVL2*, *C1orf132*/*MIR29B2C*, *FHL2*, *KLF14*, *TRIM59*), as previously reported [10,17].

The DNA samples (500 ng) were initially plated at a concentration of 25 ng/μL in 96-well plates. Sodium bisulphite conversion was performed using the EZ-96 DNA Methylation-Gold™ Kit from Zymo Research (Irvine, CA, USA), following the manufacturer’s instructions. After bisulphite treatment, the DNA was eluted to a final volume of 200 μL. To set up the PCR reaction, 10 μL of the bisulphite-treated template DNA was combined with 25 μL of GoTaq Hot Start Green Master Mix (Promega). Additionally, 1 μL of the forward primer (10 μM) and 1 μL of the 5′-end biotinylated reverse primer (10 μM) was added. This resulted in a total reaction volume of 50 μL. The PCR reaction was subjected to the following cycling conditions, which have been previously described [10], and the primer sequences and sequencing regions are reported in Supplementary Table S1. Biological (epigenetic; DNAm) age (Y) was calculated as follows:

Y = 3.26847784751817 + 0.465445549010653 methC7-*ELOVL2* − 0.355450171437202 methC1 − *C1orf132/MIR29B2C* + 0.306488541137007 methC7-*TRIM59* + 0.832684435238792 methC1-*KLF14* + 0.237081243617191 methC2-*FHL2*

### 2.5. Statistical Analysis

A descriptive analysis was conducted on the socio-demographic and clinical characteristics of the population. In cases where the data showed a normal distribution, the mean and standard deviation were reported; otherwise, the median and quartiles were reported. For qualitative variables, counts and respective percentages were provided. The degree of linear correlation between chronological age and biological age was evaluated using Pearson’s correlation coefficient. To obtain an estimate of biological aging that was independent of chronological age, a measure known as age acceleration was used. It was calculated by applying a simple linear regression model with chronological age as the independent variable and biological age as the outcome. The residual of this statistical model, which is the difference between the observed biological age and the one predicted by the model, represents the acceleration of aging due to epigenetic effects [18,19].

If biological age is greater than chronological age, the age acceleration will have a positive value, expressed in years; otherwise, it will be negative. To assess the association of the clinical and socio-demographic characteristics of the study population with age acceleration, univariate regression models were used. Then, we applied a multivariable stepwise linear regression model to identify an efficient combination of independent predictors that was significantly associated with age acceleration. Given the existence of multicollinearity among the predictor variables, the variance inflation factor (VIF) statistic was calculated, and the variables selected for entry into the multivariable stepwise model were heart rate, systolic blood pressure, total cholesterol, glycated hemoglobin, neutrophils, gender, smoking, and body mass index (BMI). Lastly, through a univariate linear regression model, it was evaluated whether age acceleration was associated with the probability of experiencing a cardiovascular event in the next ten years, measured using the FRS for cardiovascular risk. The FRS was previously transformed using the natural logarithm to satisfy the assumption of normality of the linear regression model.

All the statistical analyses were performed and the corresponding graphical representations generated using SAS 9.4 statistical software (SAS Institute Inc., Cary, NC, USA).

## 3. Results

### 3.1. Study Population

The main demographic and lifestyle characteristics of the study population are reported in Table 1. The mean age was 51.7 ± 18.1 years. Of the 190 enrolled subjects, 75.6% were women, 26.3% were overweight (BMI 25–30 kg/m^2^), 42.1% showed first-degree obesity (30 ≤ BMI < 35), and 31.6% had second-degree obesity (BMI ≥ 35 kg/m^2^). Regarding the lifestyle of the subjects enrolled, 46.3% were non-smokers and 53.7% were smokers; 42% consumed alcohol; 56% had a sedentary lifestyle; and only 5.8% defined themselves as sporty.

The clinical characteristics of the participants in the study are reported in Table 2. Among the 143 women, 84 (58.8%) were in menopause, and the mean age of menopause onset was 47.7 ± 6.2 years. Of the 190 enrolled subjects, 79 referred (41.6%) to having metabolic syndrome. Their mean systolic blood pressure was 124.2 ± 17.9 mmHg, while their mean diastolic blood pressure was 77.0 ± 9.6 mmHg, and 71 out of 179 referred to the use of antihypertensive medications. A total of 40 subjects (21.0%) suffered from diabetes, and 24 (12.6%) referred to using medication for that condition. Their mean total cholesterol concentration was 204.1 ± 41.2 mg/dL, and 26 (13.7%) used lipid-lowering medications.

### 3.2. Cardiovascular Parameters and DNAm Age

We tested the correlation between chronological age and DNA methylation (DNAm) age and observed that chronological age was positively correlated with DNAm age as estimated through the Zbieć-Piekarska method (ρ = 0.92, *p* < 0.001, R^2^ = 0.85).

The associations between demographic, lifestyle, and clinical characteristics and DNAm age acceleration were explored through univariate regression analyses, and the results are reported in Table 3. Several parameters that contribute to increased cardiovascular risk, such as systolic blood pressure (β = 0.05; SE = 0.02; *p* = 0.016), blood glucose (β = 0.03; SE = 0.01; *p* = 0.030), and glycated hemoglobin levels (β = 0.11; SE = 0.04; *p* = 0.013), as well as increased heart rate (β = 0.10; SE = 0.03; *p* = 0.003), were associated with DNAm age acceleration. In addition, individuals with diabetes exhibited an average DNAm age acceleration of 1.45 years, while non-diabetic subjects displayed a DNAm age deceleration of −0.79 years (β = 2.25; SE = 0.84; *p* = 0.008). Among the women in menopause, the average DNAm age acceleration was 1.08 years, compared to −1.86 years for the women not in menopause (β = 2.94; SE = 1.22; *p* = 0.016). Both neutrophil (β = 0.10; SE = 0.04; *p* = 0.018) and granulocyte (β = 0.09; SE = 0.44; *p* = 0.033) counts were associated with increased DNAm age acceleration, while lymphocyte count was not (β = −0.08; SE = 0.04; *p*-value = 0.068).

**Figure 1:**
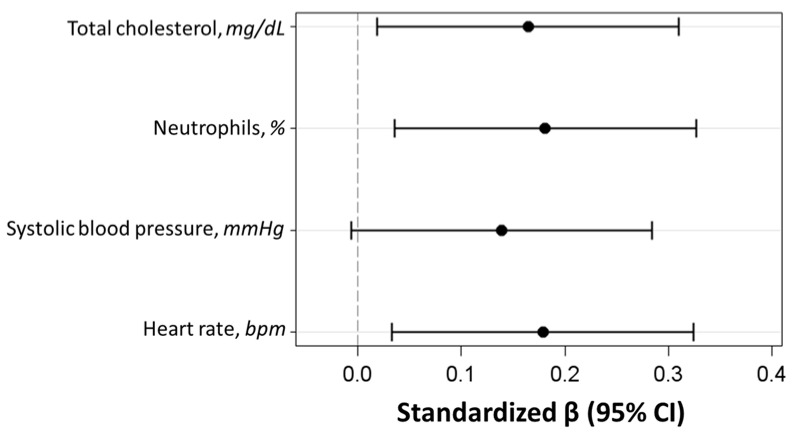
Standardized regression β coefficients with 95% confidence intervals (CIs) of the multivariable stepwise linear regression model evaluating the association of demographic, lifestyle, and clinical characteristics with DNAm age acceleration.

We applied a multivariable stepwise linear regression model to identify the combination of independent predictors significantly associated with DNAm age acceleration. We observed that heart rate, systolic blood pressure, total cholesterol, and neutrophils were positively associated with DNAm age acceleration (Table 4; Figure 1), with the neutrophil concentration in the plasma as the independent variable with the strongest association (*p* = 0.015).

### 3.3. DNAm Age Acceleration Is Associated with Cardiovascular Risk

**Figure 2:**
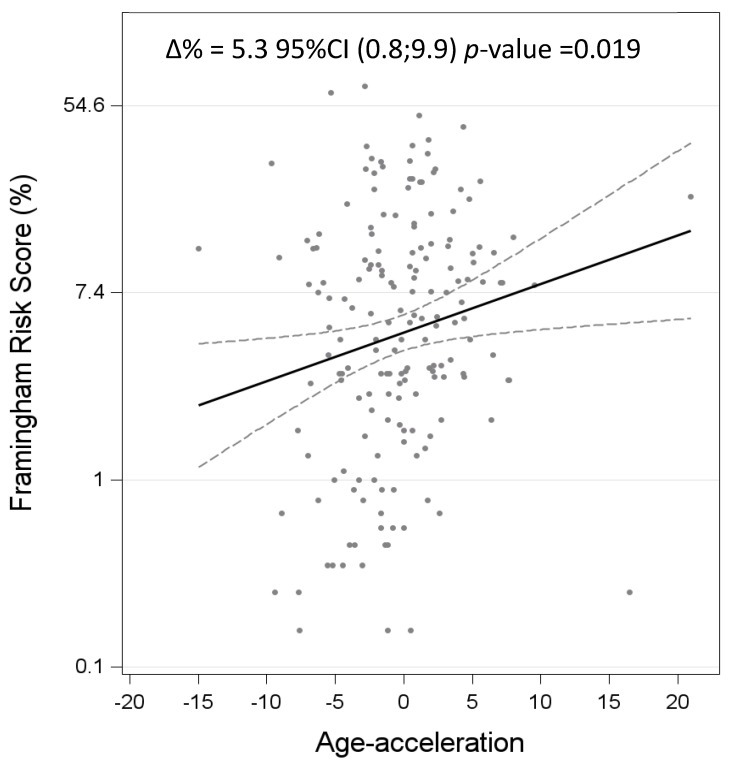
Association between DNAm age acceleration and FRS. ∆% is equal to (exp(β) − 1) × 100 and represents the percentage increase in Framingham risk score for a one-unit increase in age acceleration.

We further assessed the effects of DNAm age acceleration on the FRS and observed that each year of increase in DNAm age acceleration corresponded to an increase of 5.3% in the FRS (∆% 5.3%, 95% CI 0.8; 9.9, *p* = 0.019), as reported in Figure 2.

## 4. Discussion

In the present study, we explored the relationship between cardiovascular risk factors, such as blood glucose, diabetes, blood pressure, heart rate, and menopause, and DNAm age in a population of 190 hypersusceptible individuals with the condition of overweight/obese. Additionally, we assessed the association between DNAm age acceleration and the cardiovascular risk that was estimated by calculating the FRS.

We specifically focused on a population of subjects with the condition of overweight/obesity, as it is a major cardiovascular risk factor [17,20,21]. Our findings are in accordance with previous studies conducted in different populations of individuals in normal condition or with overweight, indicating that BMI is associated with DNAm age acceleration. In particular, Navalainen and colleagues investigated the relationship between BMI and age acceleration in three different cohorts (i.e., young adults, middle-aged, and nonagenarians) and found that BMI was correlated with increased age acceleration in middle-aged individuals [22]. Although data from a large longitudinal study conducted by Sun and colleagues suggest that a high BMI might be a cause rather than a consequence of DNA methylation changes [23], no such evidence has been reported so far to clarify whether obesity acts as a driver of or is a result of epigenetic age acceleration [24]. Several studies have delved into obesity-associated DNAm aging within metabolically active tissues [25]. These investigations have validated obesity-related DNAm age acceleration in the blood and liver, adipose, and buccal tissues.

To estimate DNAm age, we utilized the DNAm age predictor model developed by Zbieć-Piekarska and colleagues [10]. Unlike traditional epigenetic clocks, which are based on the assessment of DNAm age through a methylome-wide approach, clocks that rely on fewer CpGs (referred to as minimized clocks) offer the possibility of being scaled up or operated at a reduced sample cost. Previous studies have proposed several minimized clocks utilizing robust age-related CpGs (such as *ELOVL2* and *FHL2*) [26]. The Zbieć-Piekarska model was originally developed for forensic analyses, and it has since been adapted and validated in other studies, demonstrating a high level of accuracy in human blood samples [27]. In this epigenetic clock, the CpG sites evaluated are located in the *ELOVL2*, *C1orf132*/*MIR29B2C*, *TRIM59*, *KLF14*, and *FHL2* genes [10]. *ELOVL2* encodes an enzyme involved in the elongation of long-chain polyunsaturated fatty acids, playing a role in the regulation of aging [28,29]. *C1orf132*/*MIR29B2C*, located on 1q32.2, has been correlated with aging processes. Recently, this gene was found to encode the microRNA *hsa-miR-29b-2*, which regulates gene expression post-transcriptionally and has roles in cellular senescence and aging [10]. The *TRIM59*-encoded protein is involved in the ubiquitination and degradation of target proteins, playing a role in cellular stress responses and oncogenesis [30]. It has been associated with aging-related changes in the epigenome [31]. The *KLF14* gene encodes a transcriptional regulator implicated in metabolic processes, including glucose metabolism and lipid regulation [32]. The *FHL2* gene encodes a conserved protein involved in signal transduction, transcriptional regulation, and cytoskeletal organization, previously correlated with aging [10].

In our study population, chronological age showed a strong positive association with DNAm age. This result confirmed the reliability of the DNAm age estimator proposed by Zbieć-Piekarska and colleagues, as previously reported by other studies in different populations [17,33]. In addition to first-generation and minimized clocks, second-generation clocks such as the PhenoAge Clock incorporate clinical biomarkers to predict mortality and age-related diseases [34], while the GrimAge Clock estimates mortality risk by considering DNA methylation markers associated with lifestyle and environmental factors. A recently introduced third-generation epigenetic clock, known as the Dunedin Pace of Aging methylation clock (DunedinPoAm), tracks longitudinal changes in several biomarkers related to organ system integrity over time to measure the rate at which individuals age [35]. These clocks collectively provide nuanced perspectives on biological age and offer valuable insights into age-related diseases and mortality risk [36]. It will be crucial to validate the results obtained in the present exploratory study in future studies utilizing clocks that integrate clinical parameters into the estimation of biological age. This approach will provide a comprehensive view of cardiovascular risk factors.

We observed that menopause, systolic blood pressure, glucose and glycated hemoglobin levels, diabetes, heart rate, and neutrophil and granulocyte concentrations were associated with DNAm age acceleration. After applying a multivariable stepwise linear regression model, we further observed that heart rate, total cholesterol, and neutrophil concentration were the strongest variables associated with DNAm age acceleration. Interestingly, neutrophil concentration had the most significant association, thus supporting the involvement of inflammation. Our results are in accordance with the evidence reported by Horvath et al. 2015, who demonstrated that DNAm age acceleration assessed in the peripheral blood was associated with increased neutrophil and granulocyte counts, suggesting that this condition is linked to pathogenic mechanisms involving the activation of inflammatory processes [37]. Consistently, overweight/obesity conditions and CVDs are characterized by chronic, low-grade inflammation. Overweight or obesity conditions often exacerbate systemic metabolic dysfunction, including dyslipidemia and insulin resistance, leading to cardiometabolic syndrome, involving a constellation of metabolic abnormalities that are CVD risk factors [38,39].

Aging plays a crucial role in CVD, and older individuals are not only more prone to developing acute CVDs but also more likely to succumb to them [40]. This is due to the natural decline in tissue and cell functions with age [41]. In addition, during the aging process and in age-related diseases, inflammation plays a critical role since increased systemic inflammation, known as “inflammaging”, predisposes individuals to several age-related conditions, including cardiovascular diseases [42]. In this context, the use of epigenetic clocks has proven instrumental in estimating individuals’ biological age and exploring the aging process. Additionally, they could be powerful tools for assessing the effects of exposure to various risk factors on biological functionality through estimates of DNAm age acceleration [25]. Indeed, higher DNAm age acceleration is suggestive of tissues aging faster than expected according to chronological age and has been associated with worsening outcomes as measured by cardiometabolic risk factors [43].

Several studies have demonstrated the impact of multiple inflammatory factors on DNAm age and their association with different diseases, including CVDs, in different populations [44,45,46,47]. In light of this, we investigated the association between DNAm age acceleration and cardiovascular risk as assessed using the FRS in our population of highly susceptible individuals and observed that DNAm age acceleration increases the FRS. Previous studies have examined the link between DNAm age acceleration and the FRS in different cohorts, such as the Framingham Heart Study, the Atherosclerosis Risk in Communities, and the Cardiovascular Health Study [48,49]. However, the results remained inconclusive, likely due to heterogeneity in the study designs, the specific outcomes examined, and the different epigenetic aging measures [50,51]. A recent study demonstrated the relationship between increased DNAm age acceleration and a higher FRS in an African American population characterized by an average BMI indicative of overweight [47].

Lifestyle improvements such as diets and physical activity could reduce epigenetic age, leading to an overall improvement in health, as demonstrated by the Diet, Physical Activity, and Mammography (DAMA) study, and consequently may reduce the FRS [52].

The mean age of menopause onset in our population was lower (47.7 years) than that of the general European population (i.e., 50.5 years) [53]. We observed that menopause induced DNAm age acceleration, in accordance with the evidence previously described by Levine and colleagues in three different populations of women of a normal weight [54]. To our knowledge, this is the first study to investigate the link between menopause and age acceleration in a population of selected women with overweight or obesity. The link between overweight/obesity and the age of menopause onset is still controversial [55]; however, women who experience natural menopause at a later age have a lower risk of CVD [56]. Different mechanisms have been proposed for linking menopause and CVD, such as menopause-related hot flashes and night sweats, which might affect blood pressure [55,56]. Recent evidence shows that depression during the menopause transition is linked to a higher cardiovascular disease risk [57,58]. On the other hand, menopause onset could be precociously induced by CVD during reproductive years, cigarette smoking, or genetic factors [59]. Future studies are needed to identify the molecular mechanisms underlying the link between menopause and CVD with the aim of clarifying the causal relationship between these two conditions.

The novelty of this study lies in its demonstration of the relationship between cardiovascular risks and DNAm age acceleration within a specific population of highly susceptible individuals with overweight or obesity. Furthermore, this evidence was obtained using a parsimonious estimator of DNAm age, which could potentially allow for its large-scale application.

We acknowledge some limitations of our study. First, the study was cross-sectional, so it was not possible to determine the predictive capability of DNAm age acceleration for cardiovascular outcomes. However, it was possible to assess its relationship with specific cardiovascular risk factors. Therefore, future longitudinal studies in larger populations of both hypersusceptible and healthy subjects will be necessary to determine the predictive value of DNAm age for cardiovascular outcomes. Moreover, we used an algorithm based on a “reference population”. This introduces confounding variables that are difficult to accurately assess. Consequently, future research should prioritize the development of reliable algorithms to enhance our ability to identify individuals at risk, ultimately leading to improved control and follow-up measures for preventive interventions.

## 5. Conclusions

Our study has revealed for the first time the association between DNAm age acceleration and cardiovascular risk in individuals with overweight or obese and offers valuable insights into the relationship between BMI, DNAm age, and cardiovascular risk. The obtained evidence indicates that DNAm age might be considered a potential effect biomarker of cumulative exposure to different cardiovascular risk factors and pave the way to future prospective studies aimed at evaluating the predictive potential of epigenetic clocks for cardiovascular risk assessment.

## Figures and Tables

**Table 1:** Participant characteristics at study enrollment (N = 190).

Characteristics	
Age, *years*	51.7 ± 18.1
Gender	
*Males*	47 (24.7%)
*Females*	143 (75.6%)
BMI, kg/m^2^	33 ± 4.6
BMI < 30	50 (26.3%)
BMI 30–35	80 (42.1%)
BMI ≥ 35	60 (31.6%)
Smoking status	
*Non-smoker*	88 (46.3%)
*Smoker*	102 (53.7%)
Alcohol consumption	
*Yes*	80 (42.2%)
*No*	55 (28.9%)
*Missing*	55 (28.9%)
Physical activity levels	
*Sedentary*	108 (56.8%)
*Active*	59 (31.0%)
*Sporty*	11 (5.8%)
*Active and sporty*	6 (3.2%)
*Missing*	6 (3.2%)

BMI indicates body mass index. Continuous variables are expressed as means  ±  standard deviation (SD); discrete variables are expressed as counts (%).

**Table 2:** Clinical characteristics of participants at study enrollment (N = 190).

Clinical Characteristics	
Menopause (only for 143 women)	
*Yes*	84 (58.8%)
*No*	55 (38.5%)
*Missing*	4 (2.7%)
Metabolic syndrome	
*Yes*	79 (41.6%)
*No*	111 (58.4%)
Blood pressure, mmHg	
*Systolic*	124.2 ± 17.9
*Diastolic*	77.0 ± 9.6
Antihypertensive medications	
*Yes*	71 (37.4%)
*No*	119 (62.6%)
Glucose, mg/dL	92 [85, 103]
Glycated hemoglobin, mmol/mol	39.8 [36.6, 43]
Insulin level, U/mL	12.0 [8.8, 17.9]
Diabetes	
*Yes*	40 (21.0%)
*No*	150 (79.0%)
Diabetes medications	
*Yes*	24 (12.6%)
*No*	166 (87.4%)
Triglycerides, mg/dL	100 [75, 145]
Total cholesterol, mg/dL	204.1 ± 41.2
HDL, mg/dL	58.6 ± 15.8
LDL, mg/dL	125.2 ± 36.7
Lipid-lowering medications	
*Yes*	26 (13.7%)
*No*	164 (86.3%)
Heart rate, bpm	66.8 ± 10.2
Fibrinogen, mg/dL	334 ± 63.6
C-reactive protein, mg/L	0.30 [0.15, 0.49]
Serum creatinine, mg/dL	0.8 ± 0.2
AST, U/L	20 [17, 24]
ALT, U/L	20 [15, 30]
Gamma-flutamyltransferase, U/L	17 [12, 28]
TSH, U/mL	1.9 [1.2, 2.6]
Neutrophils, %	58.5 ± 7.9
Eosinophils, %	2.6 ± 1.6
Lymphocytes, %	30.5 ± 7.7
Monocytes, %	7.7 ± 1.9
Basophils, %	0.5 ± 0.3
Granulocytes, %	61.7 ± 7.6
Framingham risk score, %	5.8 [2.1, 12.2]

Continuous variables are expressed as means  ±  standard deviation (SD) or as medians [first quartile-third quartile] if not normally distributed; discrete variables are expressed as counts (%).

**Table 3:** Association between demographic, lifestyle, and clinical characteristics and DNAm age acceleration according to univariate linear regression models (N = 190).

	β	SE	*p*-Value	
**Gender**				
*Female vs. male*	0.997	0.791	0.209	
BMI, kg/m^2^	0.009	0.073	0.898	
*BMI 30;35 vs. BMI < 30*	1.053	0.833	0.208	0.523
*BMI ≥ 35 vs. BMI < 30*	−0.287	0.884	0.746	
Smoking habits				
*Smoker vs. non-smoker*	−0.708	0.675	0.269	
Alcohol consumption				
*Yes vs. No*	−0.198	0.752	0.793	
Physical activity levels				
*Active vs. sedentary behavior*	0.968	0.746	0.196	
*Sporty vs. sedentary behavior*	−0.927	1.475	0.531	0.619
*Active and sporty vs. sedentary behavior*	1.811	1.869	0.334	
Menopause (only women)				
*Yes vs. no*	**2.219**	**0.767**	**0.005**	
Metabolic syndrome				
*Yes vs. no*	0.875	0.688	0.205	
Blood pressure, mmHg				
*Systolic*	**0.045**	**0.019**	**0.019**	
*Diastolic*	0.036	0.035	0.303	
Antihypertensive medications				
*Yes vs. no*	0.336	0.703	0.633	
Glucose, mg/dL	**0.025**	**0.012**	**0.030**	
Glycated hemoglobin, mmol/mol	**0.105**	**0.042**	**0.013**	
Insulin level, U/mL	0.033	0.038	0.389	
Diabetes				
*Yes vs. No*	**2.247**	**0.841**	**0.008**	
Diabetes medications				
*Yes vs. no*	1.145	1.042	0.273	
Triglycerides, mg/dL	0.001	0.003	0.640	
Total cholesterol, mg/dL	0.015	0.008	0.069	
HDL, mg/dL	0.006	0.021	0.774	
LDL, mg/dL	0.013	0.009	0.173	
Lipid-lowering medications				
*Yes vs. no*	0.896	1.022	0.382	
Heart rate, bpm	**0.096**	**0.032**	**0.003**	
Fibrinogen, mg/dL	−0.001	0.005	0.904	
C-reactive protein, mg/L	0.175	0.492	0.723	
Serum creatinine, mg/dL	−0.327	1.526	0.831	
AST, U/L	−0.013	0.035	0.713	
ALT, U/L	−0.011	0.014	0.434	
Gamma-glutamyltransferase, U/L	0.002	0.016	0.922	
TSH, U/mL	0.295	0.255	0.249	
Neutrophils, %	**0.100**	**0.042**	**0.018**	
Eosinophils, %	−0.252	0.207	0.225	
Lymphocytes, %	−0.080	0.043	0.068	
Monocytes, %	−0.150	0.183	0.416	
Basophils, %	−0.483	1.149	0.674	
Granulocytes, %	**0.095**	**0.044**	**0.033**	

The significant variables (*p*-value ≤ 0.05) are reported in bold.

**Table 4:** Association of demographic, lifestyle, and clinical characteristics with DNAm age acceleration by multivariable stepwise linear regression model (N = 190).

	β	SE	95% CI	Partial Correlation Coefficient	*p*-Value
Heart rate, bpm	**0.078**	**0.032**	**(0.014; 0.141)**	**0.183**	**0.016**
Systolic blood pressure, mmHg	0.035	0.018	(−0.002; 0.071)	0.143	0.061
Total cholesterol, mg/dL	**0.019**	**0.008**	**(0.020; 0.185)**	0.168	**0.028**
Neutrophils, %	**0.102**	**0.041**	**(0.002; 0.035)**	**0.185**	**0.015**

The significant values (*p*-value ≤ 0.05) are reported in bold.

## Data Availability

The data that support the findings of this study are available from the corresponding author upon reasonable request.

## Supplementary Materials

The following supporting information can be downloaded at https://www.mdpi.com/article/10.3390/biomedicines12081631/s1. Table S1: Oligo sequences and sequenced regions for methylation analysis.
