# Supplementary material for: Investigating the Relationship between Epigenetic Age and Cardiovascular Risk in a Population with Overweight/Obesity
Source: Biomedicines. 2024 Jul 23;12(8):1631. doi: 10.3390/biomedicines12081631 (PMC11351200; doi:10.3390/biomedicines12081631)
Supplement: Supplementary file 1 [file biomedicines-12-01631-s001.zip › biomedicines-3090188-supplementary.pdf]

Supplementary Material

Supplementary Table S1

GENE	FORWARD OLIGO (5'-3')	REVERSE OLIGO (5'-3')	SEQUENCING PRIMER (5'-3')	ANALYZED SEQUENCE
C1orf132	Biotin-GAGAAAGAAGGTGAGAAAGATAGA	AAAACCAAATTCTAAAACATTC	AAACCAAAATTTAAATC	TACA/GCAAACA/GACA/GATAAATAATCC
ELOVL2	Biotin-GGAGGGGC/TGTAGGGTAAGTGA	AACAAAACCATTTCCCCCTAATA	AATAAATATTCCTAAAACT	CCA/GTAAACA/GTTAAACCA/GCCA/GCA/GCA/GAAACCA/GAC
FHL2	GGGTTTTGGGAGTATAGTAGTTA	Biotin-AAAATAACCCCCTCCTCCCT	TTTTGGGAGTATAGTAGTTA	TC/TGGGAGC/TGTC/TGTTTTC/TGGC/TGTGGGTTTTC/TGGGC/TGC/TGAGTTTC/TGGAC/TGAGGT
KLF14	TGGTTAAGTTATGTTTAATAGTTTTA	Biotin-AACTACTACAACCCAAAAATTCC	TTTTAGAAATTATTTTGTTT	TC/TGC/TGTTTTTTTTTTTGTC/TGGC/TGAG
TRIM59	GGTTTTTTATTTTGTGGGGAGT	Biotin-TCCAAAACCCCTTCTCCTATACT	GGAGAGGTTGGGTTTG	GC/TGC/TGGGAC/TGAGGC/TGAAGC/TGTC/TGGTGGTC/TGAC/TGGTTT
